# Supplementary material for: Gender-Dependent Deregulation of Linear and Circular RNA Variants of HOMER1 in the Entorhinal Cortex of Alzheimer’s Disease
Source: Int J Mol Sci. 2021 Aug 26;22(17):9205. doi: 10.3390/ijms22179205 (PMC8430762; doi:10.3390/ijms22179205)
Supplement: Supplementary file 1 [file ijms-22-09205-s001.zip › supplemental caption figures and tables.pdf]

Supplemental Figure S1: *HOMER1* RNA variants expression. A) Box-plot represent the percentage of *HOMER1B/C* and *HOMER1A* expression relative to geometric mean of *GAPDH* and *ACTB* housekeeping genes expression in total cohort (n=44).

Supplemental Figure S2: *HOMER1* linear RNA and circRNA expression across AD stage in female group. A) Box-plot represent the percentage of *HOMER1* and *circHOMER1* RNAs expression according to the ABC score classification relative to geometric mean of *GAPDH* and *ACTB* housekeeping genes expression. Control, n=5; Low, n=6; Middle, n=8, High, n=3. B) Box-plot represent the percentage of *HOMER1* and *circHOMER1* RNA expression according to the Braak and Braak stage classification relative to geometric mean of GAPDH and ACTB housekeeping genes expression. Control, n=5; I-II, n= 5; III-IV, n= 9; V-VI, n=3. *p value < 0.05; **p value < 0.01.

Supplemental Figure S3: The decrease in expression of the different *HOMER1* variants is proportional within AD female cases. A) The box-plot represent the differences in relative expression between the different *HOMER1* variants. B) Representation proportional decreased of log(relative expression) of each *HOMER1* variant in control and AD cases.

Supplemental Figure S4: HOMER1 B/C protein decreased in human entorhinal cortex in Alzheimer´s disease. A) Western blot of HOMER1 B/C protein shows two bands, both bands were taken into account to perform the analysis of WB results. Human entorhinal cortex samples from controls and AD cases were loaded as labeled at the top of the lanes, the box plot represents the HOMER1 B/C /GAPDH ratio.

Supplemental Table S1: Brain sample set characteristics. The table shows the characteristic of the samples included in the study. No.: Number; ABC score: NPD: no protein deposit; h: hours; AD: Alzheimer's disease; PMI: post mortem interval.

Supplemental Table S2: Summary of age and gender characteristics of subjects included in the study.
