# Supplementary material for: Gender-Dependent Deregulation of Linear and Circular RNA Variants of HOMER1 in the Entorhinal Cortex of Alzheimer’s Disease
Source: Int J Mol Sci. 2021 Aug 26;22(17):9205. doi: 10.3390/ijms22179205 (PMC8430762; doi:10.3390/ijms22179205)
Supplement: Supplementary file 1 [file ijms-22-09205-s001.zip › supplemental Table S1.pdf]

Nº	Braak stage	ABC score	ABC scale	% β- amiloid plaque area	Gender	Age at death (years)	PMI (h)
1	III	A1B2C1	Low	0,349	Female	96	1,5
2	III	A1B2C1	Low	0,044	Female	88	33
3	I	A1B1C1	Low	0.00	Female	85	4,3
4	NA	NA	NA	0,672	Female	69	NA
5	II	A1B1C1	Low	0,147	Female	66	1,4
6	III	A1B2C3	Int	0,648	Female	84	13
7	IV	A2B2C2	Int	0,814	Female	97	NA
8	IV	A2B2C3	Int	0,420	Male	78	5
9	I	A1B1C1	Low	0,000	Male	60	15,3
10	V	A3B3C2	High	NA	Male	91	5
11	III	A3B2C3	Int	0,724	Male	83	9
12	IV	A3B2C1	Int	0,131	Female	90	3
13	I	A1B1C1	Low	0,024	Male	85	3,2
14	III	A3B2C3	Int	0,376	Female	85	NA
15	III-IV	A3B2C3	Int	0,357	Female	98	3
16	IV	A2B2C2	Int	0,431	Female	91	10
17	III	A2B2C3	Int	0,528	Female	98	23
18	V	A3B3C3	High	0,777	Female	77	11
19	VI	A3B3C3	High	0,777	Female	86	2,3
20	V	A3B3C2	High	1,610	Female	82	9
21	I	A1B1C1	Low	NA	Female	85	2
22	IV	A2B2C2	Int	0,959	Male	88	3,5
23	VI	A3B3C3	High	2,013	Male	70	2,35
24	VI	A3B3C3	High	1,545	Male	59	4
25	II	A2B1C3	Low	NA	Male	80	3
26	II	A1B1C1	Low	NA	Male	74	2,5
27	II	A1B2C2	Int	NA	Female	71	4
28	II	A2B1C1	Low	NA	Female	80	3,7
29	0	control	Not	NPD	Female	43	3
30	0	control	Not	NPD	Male	54	18
31	0	control	Not	NPD	Female	19	NA
32	0	control	Not	NPD	Female	46	7
33	0	control	Not	NPD	Male	28	6
34	0	control	Not	NPD	Male	41	3,5
35	0	control	Not	NPD	Male	54	2,7
36	0	control	Not	NPD	Male	81	10,5
37	0	control	Not	NPD	Male	26	6,2
38	0	control	Not	NPD	Male	53	7
39	0	control	Not	NPD	Female	88	9
40	0	control	Not	NPD	Male	66	6,5
41	0	control	Not	NPD	Female	88	3,5
42	0	control	Not	NPD	Female	76	11,5
43	0	control	Not	NPD	Male	65	3
44	0	control	Not	NPD	Male	83	NA
