# Supplementary material for: Gender-Dependent Deregulation of Linear and Circular RNA Variants of HOMER1 in the Entorhinal Cortex of Alzheimer’s Disease
Source: Int J Mol Sci. 2021 Aug 26;22(17):9205. doi: 10.3390/ijms22179205 (PMC8430762; doi:10.3390/ijms22179205)
Supplement: Supplementary file 1 [file ijms-22-09205-s001.zip › supplemental Table S2.pdf]

	Controls, n= 16	AD cases, n= 28	P-value*
**Age at death, mean ± SD**	56.94 ± 5.602	82 ± 2.026	0.0001
**Gender, female %**	31.25%	64.28%	0.035

* Between controls and AD cases
