# Supplementary figures and images for: Gender-Dependent Deregulation of Linear and Circular RNA Variants of HOMER1 in the Entorhinal Cortex of Alzheimer’s Disease
Source: Int J Mol Sci. 2021 Aug 26;22(17):9205. doi: 10.3390/ijms22179205 (PMC8430762; doi:10.3390/ijms22179205)

A

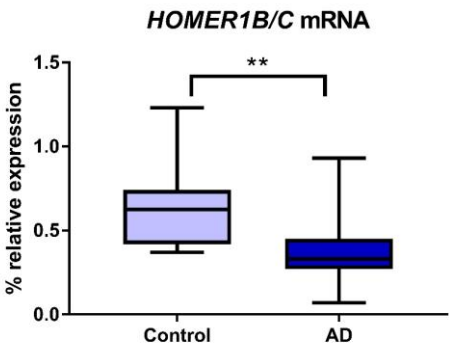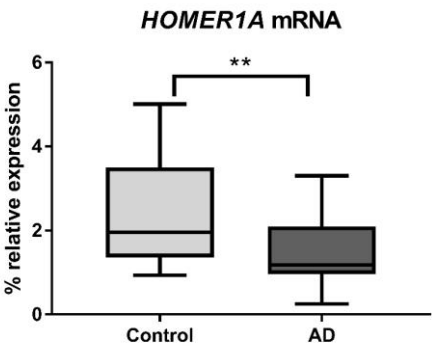

Supplement: Supplementary file 1 [file ijms-22-09205-s001.zip › supplemental Figure S1.pdf]

A

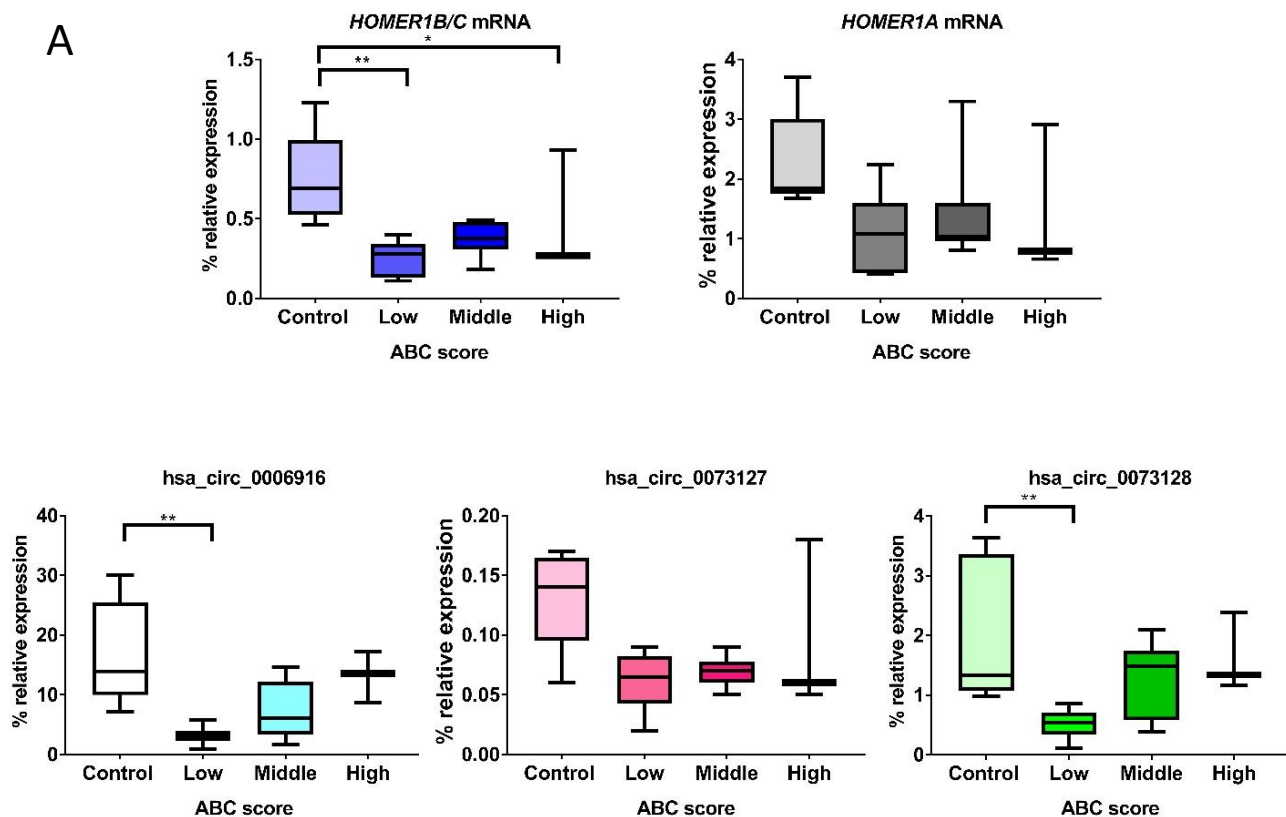

B

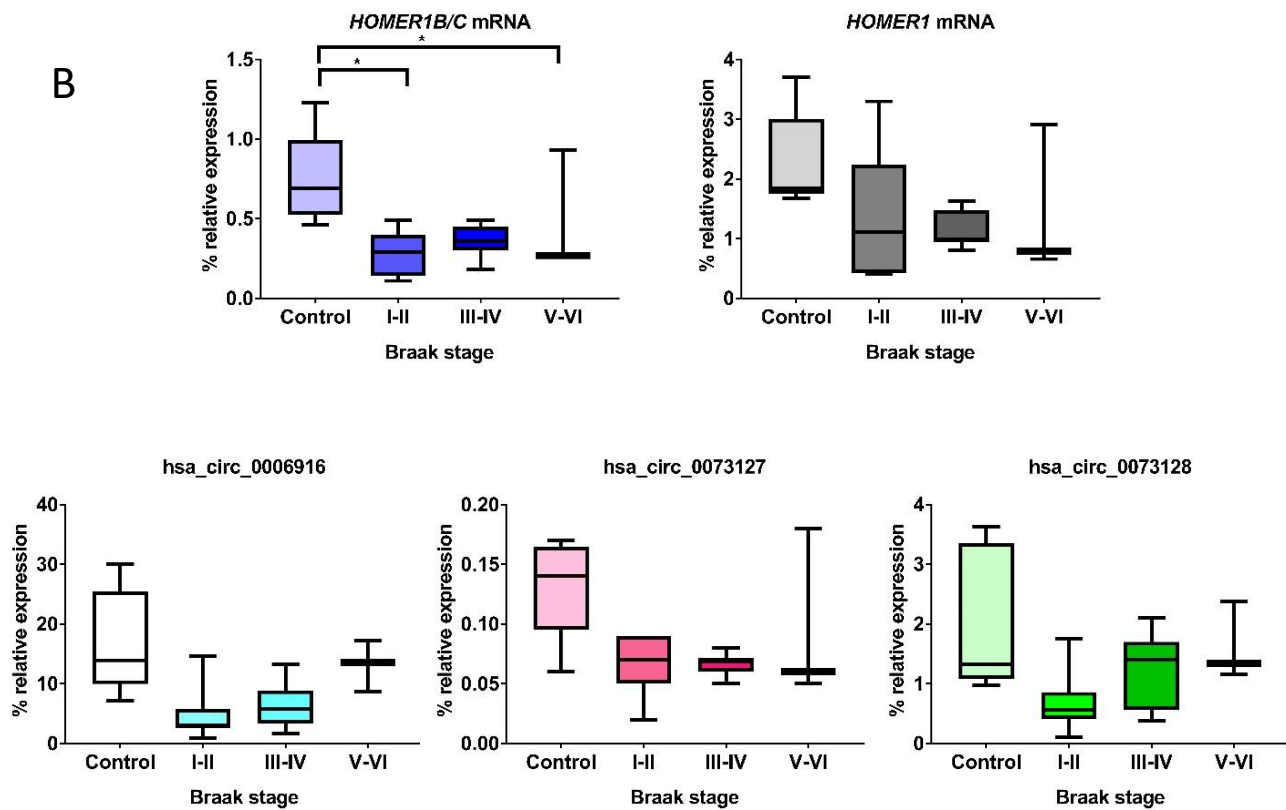

Supplement: Supplementary file 1 [file ijms-22-09205-s001.zip › supplemental Figure S2.pdf]

A

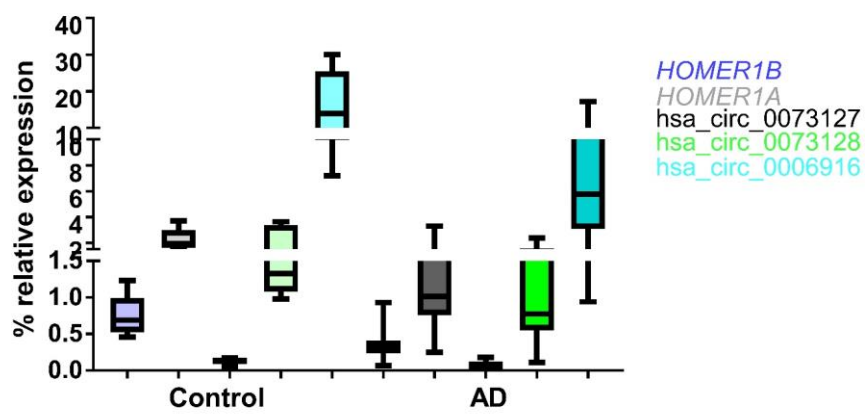

B

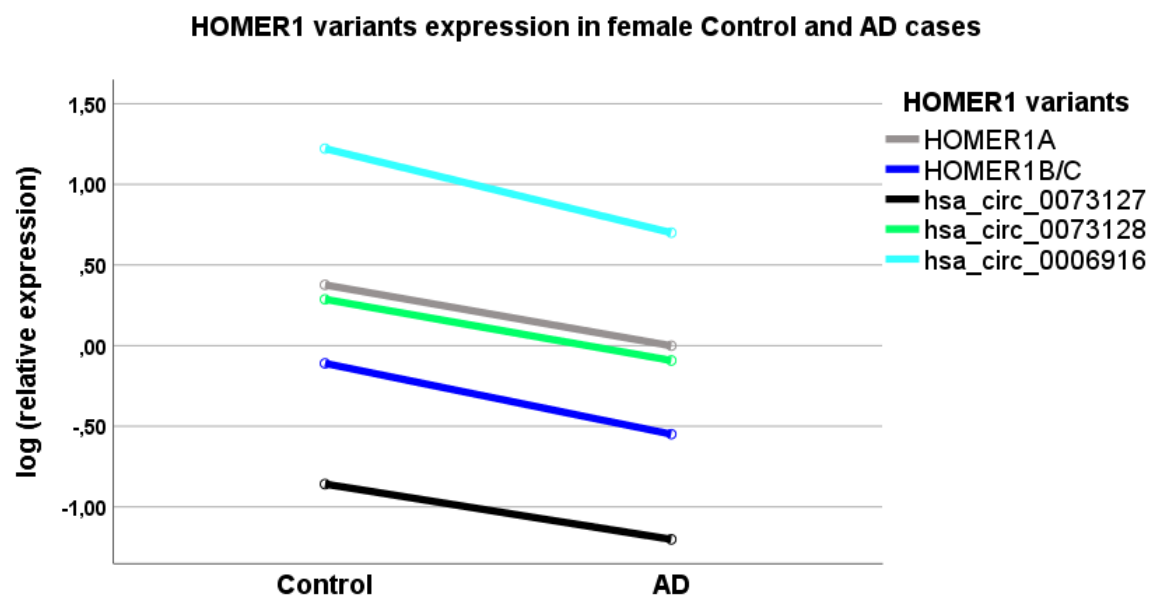

Supplement: Supplementary file 1 [file ijms-22-09205-s001.zip › supplemental Figure S3.pdf]

A

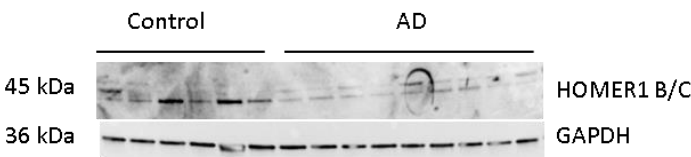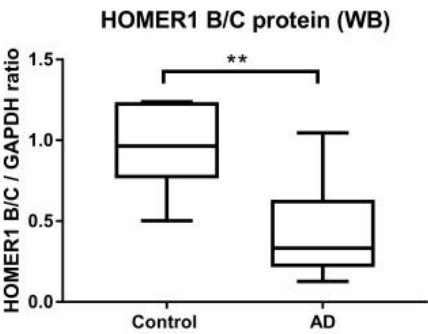

Supplement: Supplementary file 1 [file ijms-22-09205-s001.zip › supplemental Figure S4.pdf]
